# Authors’ Response to Peer Reviews of “Detecting Substance Use Disorder Using Social Media Data and the Dark Web: Time- and Knowledge-Aware Study”

**DOI:** 10.2196/57838

**Published:** 2024-05-01

**Authors:** Usha Lokala, Orchid Chetia Phukan, Triyasha Ghosh Dastidar, Francois Lamy, Raminta Daniulaityte, Amit Sheth

**Affiliations:** 1 Department of Computer Science and Computer Engineering, Artificial Intelligence Institute, University of South Carolina, Columbia, SC, United States; 2 Department of Computer Science and Engineering, Indraprastha Institute of Information Technology, Delhi, India; 3 Department of Computer Science and Engineering, Birla Institute of Technology & Science Pilani, Hyderabad, India; 4 Department of Society and Health, Mahildol University, Salaya, Thailand; 5 College of Health Solutions, Institute for Social Science Research, Arizona State University, Phoneix, AZ, United States

**Keywords:** opioid, substance use, substance use disorder, social media, US, opioid crisis, mental health, substance misuse, crypto, dark web, users, user perception, fentanyl, synthetic opioids, United States

*This is the authors’ response to peer-review reports for “Detecting Substance Use Disorder Using Social Media Data and the Dark Web: Time- and Knowledge-Aware Study.”*

## Round 1 Review

### Responses to Reviewers’ Comments

The below includes the comments from reviewers and the authors’ responses to each comment. We thank all the reviewers for their suggestions and comments.

#### Reviewer O [1]

*1. The study [2] acknowledges the challenges of crawling crypto markets and the restricted crawling process, which limits the data available for analysis.*

*Need to explain in the manuscript.*

Response: The manuscript is revised as per this comment. Crawling crypto markets poses a significant challenge when applied to data science and machine learning to study the opioid epidemic due to the restricted crawling process. However, we made our proposed data set available to the research community for further analysis.

*2. The study proposes building an opioid drug social media knowledge graph (ODSM-KG) but does not provide details on the potential impact or implications of such a graph.*

*Need to provide the details in the manuscript.*

Response: The manuscript is revised as per this comment. The impact of the ODSM-KG is described in the manuscript as follows: “To identify the approaches for mitigating the misuse of opioids, it is imperative to study consumption patterns at the national and regional levels, the influences of the pharmaceutical industry, and the sociopolitical determinants that affect consumption. Our ODSM-KG aims to create a web-based tool that will allow for the depiction of historical patterns and enable comparisons between opioids, time periods, and areas within the United States. Given that our ODSM-KG was primarily based on data, we aim to enhance its accuracy and effectiveness by seeking guidance from a subject matter domain expert. This will enable us to customize it for unique scenarios and cater to the needs of specific users.”

*3. The study explicitly states that it does not make any clinical diagnosis or treatment suggestions, which indicates a gap in translating the research findings into practical applications for addressing substance use disorder (SUD).*

*Need to justify how this study will be helpful for clinical situations.*

Response: The manuscript is revised as per this comment. While the study may not directly provide clinical diagnosis or treatment suggestions, its findings can still be highly valuable for informing clinical practice and interventions for SUD in the identification of risk factors. Even if the study does not prescribe specific treatments, its findings can inform the development of novel intervention strategies or the optimization of existing ones. For example, as our paper highlights certain sentiment and emotion patterns associated with SUD, clinicians can incorporate this knowledge into their approaches. This research also contributes to understanding the factors contributing to the onset of SUD, which can help in designing prevention programs aimed at at-risk populations, such as individuals with mental health disorders. Research findings can inform policy makers about the effectiveness of current strategies and the need for adjustments in regulations, health care policies, or resource allocation for addressing SUD at a broader level.

#### Anonymous [3]

*1. The paper is well written and easy to understand. See comments below for a summary description of the paper from my perspective.*

*2. However, I would have liked to see insights ideally established in the medical literature and supported by the experimental context in this paper (eg, those that can substantiate the prediction results and how this type of artificial intelligence can benefit SUD-related outcomes).*

Response: We provided a comprehensive review of relevant studies and findings from the literature that support our approach and the predictive results obtained through our artificial intelligence methodology. The experimental context is supported by the literature described in our related work. This also supports our insights and prediction results as per the literature. We discussed the study’s potential impact on how artificial intelligence can benefit SUD prediction in the Discussion section, the Global Relevance section, and the Results section.

*3. Although a temporal pattern–aware method is implemented in this paper, which is a big positive, I would like to see an analysis over two distinctly separate time periods to establish the consistency and robustness of the proposed approach.*

Response: Figures 4-6 show the consistency of sentiments, topics, and emotions for drugs since 2015. The time period considered is between 2015 and 2020 with a side-by-side comparison of drugs.

#### Anonymous [4]

*This study can be considered for publication if the researchers are able to revise it to improve the clarity of the objective, theoretical relevance, and practical value. I will suggest that there should be a segment on the objective of the study immediately after the introduction. This will help in giving the study a direction. Please, include this citation:*

*Obosi AC, Fatunbi AM, Oyinloye O. Peer pressure and substance use as predictors of mental health among in-school adolescents in Nigeria. Ianna J Interdisciplinary Stud. 2022;4(1):1‐9.*

*Explain the implication of your findings to other countries, that is, give the study an international outlook.*

Response: This paper by Obosi et al is mentioned and cited in the related work sections of the paper. Thank you for your suggestion. A section named “Global Relevance” has been added to the paper now as per your comment, and the impact is described.

## Abbreviations

ODSM-KG: opioid drug social media knowledge graph
SUD: substance use disorder
